# Attention Modulates the Auditory Cortical Processing of Spatial and Category Cues in Naturalistic Auditory Scenes

**DOI:** 10.3389/fnins.2016.00254

**Published:** 2016-06-07

**Authors:** Hanna Renvall, Noël Staeren, Claudia S. Barz, Anke Ley, Elia Formisano

**Affiliations:** ^1^Department of Cognitive Neuroscience, Faculty of Psychology and Neuroscience, Maastricht UniversityMaastricht, Netherlands; ^2^Department of Neuroscience and Biomedical Engineering, Aalto University School of ScienceEspoo, Finland; ^3^Aalto Neuroimaging, Magnetoencephalography (MEG) Core, Aalto UniversityEspoo, Finland; ^4^Institute for Neuroscience and Medicine, Research Centre JuelichJuelich, Germany; ^5^Department of Psychiatry, Psychotherapy and Psychosomatics, Medical School, RWTH Aachen UniversityAachen, Germany; ^6^Maastricht Center for Systems Biology (MaCSBio), Maastricht UniversityMaastricht, Netherlands

**Keywords:** auditory scene analysis, auditory cortex (AC), fMRI BOLD, magnetoencephalography (MEG), auditory attention

## Abstract

This combined fMRI and MEG study investigated brain activations during listening and attending to natural auditory scenes. We first recorded, using in-ear microphones, vocal non-speech sounds, and environmental sounds that were mixed to construct auditory scenes containing two concurrent sound streams. During the brain measurements, subjects attended to one of the streams while spatial acoustic information of the scene was either preserved (stereophonic sounds) or removed (monophonic sounds). Compared to monophonic sounds, stereophonic sounds evoked larger blood-oxygenation-level-dependent (BOLD) fMRI responses in the bilateral posterior superior temporal areas, independent of which stimulus attribute the subject was attending to. This finding is consistent with the functional role of these regions in the (automatic) processing of auditory spatial cues. Additionally, significant differences in the cortical activation patterns depending on the target of attention were observed. Bilateral planum temporale and inferior frontal gyrus were preferentially activated when attending to stereophonic environmental sounds, whereas when subjects attended to stereophonic voice sounds, the BOLD responses were larger at the bilateral middle superior temporal gyrus and sulcus, previously reported to show voice sensitivity. In contrast, the time-resolved MEG responses were stronger for mono- than stereophonic sounds in the bilateral auditory cortices at ~360 ms after the stimulus onset when attending to the voice excerpts within the combined sounds. The observed effects suggest that during the segregation of auditory objects from the auditory background, spatial sound cues together with other relevant temporal and spectral cues are processed in an attention-dependent manner at the cortical locations generally involved in sound recognition. More synchronous neuronal activation during monophonic than stereophonic sound processing, as well as (local) neuronal inhibitory mechanisms in the auditory cortex, may explain the simultaneous increase of BOLD responses and decrease of MEG responses. These findings highlight the complimentary role of electrophysiological and hemodynamic measures in addressing brain processing of complex stimuli.

## Introduction

Overlapping voices, a phone ringing at the background: The auditory signal at our ears usually comprises sounds from several sources. Segregation of a complex sound mixture is a magnificent example of the automatic computational capabilities of our auditory system, likely determined by the interplay between bottom-up processing of the spectral and temporal acoustic elements of the mixture and attentive selection and enhancement of the relevant sounds (Bregman, 1990).

A relevant part of auditory scene analysis relates to processing of spatial information of the sound sources. Vertical and horizontal localization of sounds relies on the direction-dependent modifications of the spectral sound profile, generated by the outer ear and head, and on the timing and sound intensity differences between the ears, respectively; perception of sound motion depends on the dynamic changes of these cues. On the basis of extensive line of studies in primates (e.g., Romanski et al., 1999; Recanzone et al., 2000; Tian et al., 2001; Lomber and Malhotra, 2008; Miller and Recanzone, 2009), a dorsal auditory stream specialized for processing of spatial information has been suggested. Neuroimaging studies in humans generally support this hypothesis. Results on sound localization (Alain et al., 2001; Warren and Griffiths, 2003; Barrett and Hall, 2006; Altmann et al., 2007) and sound motion (Baumgart et al., 1999; Lewis et al., 2000; Pavani et al., 2002; Warren et al., 2002, 2005; Hart et al., 2004; Krumbholz et al., 2005a,b; Getzmann and Lewald, 2010) suggest the involvement of posterotemporal and temporoparietal regions, especially when subjects are actively engaged in sound localization tasks (Zatorre et al., 2002). Furthermore, studies using magnetoencephalography (MEG) and functional magnetic resonance imaging (fMRI) in humans (Salminen et al., 2009; Derey et al., 2016) are consistent with studies in animals (Stecker et al., 2005; Miller and Recanzone, 2009) suggesting the existence of population rate coding in (posterior) auditory areas involved in spatial processing, with populations of neurons broadly tuned to locations in the left and right auditory spatial hemifields.

Postero-temporal auditory regions, however, are not exclusively involved in the spatial analysis of sounds. For example, activation of the planum temporale (PT) has been suggested to reflect integration of spatial and auditory object information, rather than spatial processing *per se* (Zatorre et al., 2002). In addition, manipulating the number of auditory objects within an auditory scene modifies the activation at PT similarly to spatial manipulations (Smith et al., 2010). Furthermore, task-modulated processing of sound location and identity has been demonstrated in the human non-primary auditory cortex (Ahveninen et al., 2006, 2013), suggestive of fine-grained top-down effects on extracting auditory information in real-life.

Knowledge of the auditory scene analysis in the human cortex has mainly been derived from studies applying stimuli with highly-controlled physical properties, necessary to reveal the different stages of processing. Here we take an approach toward natural auditory processing, by examining cortical processing of realistic auditory “mini-scenes” with interspersed spatial cues and different sound attributes. Using ear-insert microphones, vocal, and environmental sounds were recorded and subsequently digitally superimposed. During MEG and fMRI measurements, subjects listened to binaural mini-scenes that either did or did not preserve the original spatial aspects of the sounds (stereophonic vs. monophonic sounds). We then manipulated the top-down processing of the auditory scenes, by directing the subjects' attention either to the voice or environmental excerpts within the sounds while keeping the stimuli unchanged. This design allowed us to examine the relation between the cortical mechanisms of analyzing spatial cues and selecting sound objects from a real-life like scene.

As MEG and fMRI differ in their sensitivity to the underlying neuronal activity in complex auditory (Renvall et al., 2012a) and cognitive tasks (Vartiainen et al., 2011), both imaging modalities were applied in the present study for optimal coverage and for studying the possible discrepancies between the electrophysiological and hemodynamic measures. For example, an earlier MEG study suggested monophonic sounds to elicit stronger auditory cortical activation than pseudostereophonic sounds (Ross et al., 2004), possibly related to more synchronous neuronal activation during monophonic than pseudo-stereophonic sound presentation tracked with MEG. Furthermore, (local) neuronal inhibition in the auditory cortex has been suggested to affect coding of spatial auditory cues (e.g., Fitzpatrick et al., 1997). Thus an increase of local inhibition could lead to simultaneous increase of blood-oxygenation-level-dependent (BOLD) fMRI responses and decrease of MEG responses.

## Materials and methods

### Subjects

We studied, with written informed consent, 10 subjects with MEG (mean ± SEM age 30 ± 1 yrs; four females; nine right-handed and one ambidextrous) and 10 subjects with fMRI (28 ± 4 yrs; four females; nine right-handed and one ambidextrous). Six of the subjects participated in both MEG and fMRI studies. None of the subjects had a history of hearing or neurological impairments, and they were all naïve to the experimental setup. The study received a prior approval by the Ethical Committee of the Faculty of Psychology, University of Maastricht, The Netherlands.

### Experimental stimuli

**Figure 1:**
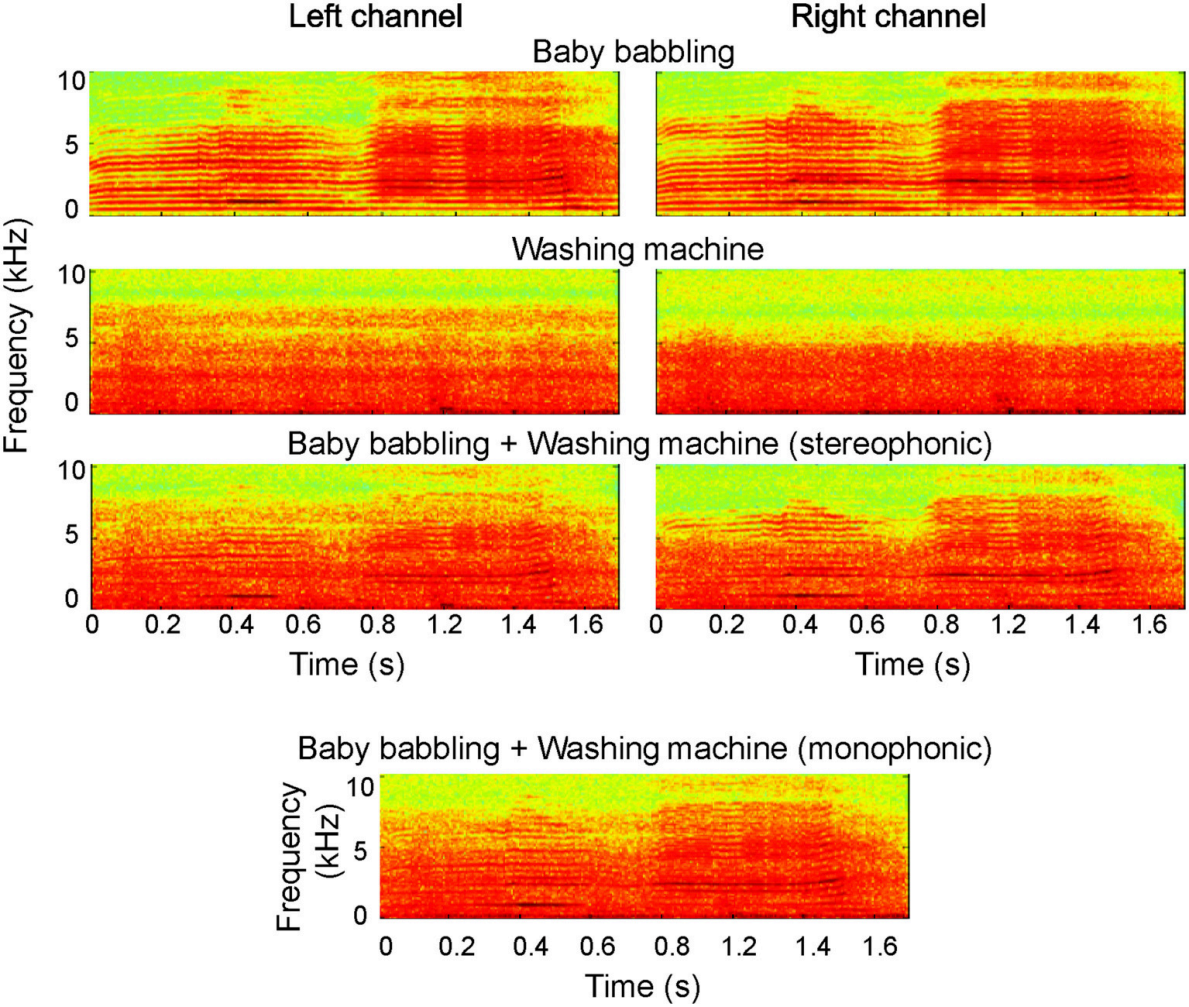
**Examples of the experimental stimuli**. The original sounds (“baby babbling” and “Washing machine”) are depicted for both left and right auditory channel (top); the combined sounds used in the experiment, in both stereophonic and monophonic forms, are shown below.

High-quality sounds were recorded binaurally using two in-ear microphones (FG-23652-P16, Knowles Electronics, Itasca, Illinois, U.S.A.) and a portable digital recorder (96 kHz, 24-bit, M-Audio MicroTrack 24/96 Pocket Digital Recorder). After recording, sounds were down-sampled to 44.1 kHz/16-bit and low-pass filtered at 10 kHz using Adobe Audition (Adobe Systems, Inc., CA, USA). The environmental sounds comprised, e.g., sounds of tool use, office sounds, and household sounds. The vocalizations were non-speech sounds produced by 12 individuals, and they comprised, e.g., laughing, coughing, sighing, and baby crying. All sounds were fairly stationary, whereas the original vocal sounds had less variability in their degree of channel separation than the environmental sounds (channel intensity difference divided by the summed intensity across channels 0.20 ± 0.02 for voices, 0.38 ± 0.02 for environmental sounds). All original sounds were reported by the participants to be very natural-like and have a clear stereophonic effect. Fifty-nine different stereophonic combinations of environmental and vocal sounds were created by mixing—keeping the two channels separate—recording excerpts that included sounds from 59 different environments and 59 vocal sounds. Before mixing, the waveforms of all original stimuli were first carefully visually inspected and edited/cut in order to have a clear signal onset. Then, after superimposing the vocal and environmental sounds, 25-ms rise times were imposed to the combined sounds to further equalize the onsets amplitude-wise. Finally the average root-mean-square levels of the sounds were matched using MATLAB 7.0.1 (The MathWorks, Inc., Natick, MA, USA). The duration of the sounds varied between 450 and 2635 ms (mean length ± SD 1306 ± 565 ms) including 25-ms rise and fall times. Examples of the original stimuli and their combinations used in the experiment (monophonic and stereophonic) are presented in Figure 1 (see also Supplementary Files 1–6).

All stimuli in the study were recorded by inserting the microphones to the ear canal of three listeners that did not take part in the fMRI nor MEG measurements. It is known that—because of inter-individual differences in head and external ear shape—non-individualized recordings as used in this study do not produce as good perceptual quality as individualized recordings or use of individualized head-related transfer functions (HRTF). However, well-localized perception has been shown to be relatively independent of the details of HRTFs (Kulkarni and Colburn, 1998), and thus we chose not to record the stimuli individually because of the difficulty of recreating similar, natural complex scenes for each subject. Indeed, each subject reported a clear and natural spatial perception of the stimuli. Given the nature of our recordings, the stimuli contained—in the *Stereophonic* condition—many spatial cues including interaural time and level differences, as well as spectral cues. While this prevents us from making any specific claim on the processing of specific cues, it does reflect auditory scene analysis in naturalistic contexts, where all cues are in fact combined.

The created stereophonic sounds were utilized in the “*Stereophonic*” experimental condition; monophonic version of the same scenes (“*Monophonic”* condition) was created by merging the two audio channels. As demonstrated in Figure 1, the overall physical characteristics remained very similar after the transformation. During the experiments, the sounds were delivered to the subjects binaurally at a comfortable listening level through plastic tubes and ear pieces (MEG: ADU audio stimulator, KAR Audio, Helsinki, Finland) or via headphones (fMRI; Commander XG, Resonance Technology, Northridge, CA).

As the behavioral responses collected during the brain measurements (see below) were too few for making statistical inferences between conditions, the stimuli were behaviorally tested with two tasks in 16 subjects outside the fMRI/MEG experiment. In the first task, the sounds were presented alone in a random order. The subjects were asked to respond with a button press, as fast and accurately as possible, whether the presented sound was a voice or environmental sound. In the second task, the stimuli were the superimposed sounds used in the actual neuroimaging experiments; the stereophonic and monophonic sounds were used in different test runs. The subjects were instructed to attend to either the voice or environmental sound excerpt of the combined sound and respond with a button press in case the attended sound part was repeated. The order of runs (mono- or stereophonic, attention to voice or environmental sound) was counterbalanced across subjects.

### FMRI experiment and signal analysis

A 2 × 2 block design with auditory space (“*Stereophonic*” vs. “*Monophonic*”) and task (attention to the voice excerpt in the combined sound, “*Voice*,” vs. attention to the environmental sound excerpt, “*Environment*”) as factors was used. The experiment consisted of two functional runs during which auditory scenes from the four different conditions were presented in a block design. Each of the runs (22 min each) included nine blocks per condition and four target blocks (see below); the sequence of conditions was randomized. Each block consisted of four TRs (TR = 4640 ms, total block duration 18.5 s), and one auditory mini-scene was presented for each TR. The blocks were separated by a fixation period of three TRs. Every block was preceded by a cue presented at the fixation point, indicating the attention condition (“*Voice*” or “*Environment*”). Subjects were instructed to respond with a button press if the attended sound part was the same in two consecutive auditory scenes. This occurred in 10% of the cases (“target blocks”; altogether two target blocks per condition and four target blocks per run). The response hand was alternated across subjects. Imaging was performed with a three Tesla Siemens Allegra (head setup) at the Maastricht Brain Imaging Center. In each subject, two runs of 282 volumes were acquired with a T2^*^-weighted gradient-echo planar imaging (EPI) sequence (TR = 4640 ms, voxel size = 2.5 × 2.5 × 2.5 mm^3^, TE = 30 ms, FOV 256 × 256; matrix size 96 × 96, 32 slices covering the cortex). Anatomical images (1 × 1 × 1 mm^3^) were collected between the functional runs using a 3D-MPRAGE T1-weighted sequence. To reduce the effect of scanner noise, the sounds were presented during silent periods using a clustered volume EPI technique that allowed for presentation of auditory stimuli in silence between subsequent volume acquisitions (van Atteveldt et al., 2004; Riecke et al., 2007; Staeren et al., 2009).

Functional and anatomical images were analyzed with BrainVoyager QX (Brain Innovation, Maastricht, The Netherlands). Pre-processing consisted of slice scan-time correction (using sinc interpolation), linear trend removal, temporal high-pass filtering to remove nonlinear drifts of seven or less cycles per time course, and three-dimensional motion correction. Temporal low-pass filtering was performed using a Gaussian kernel with FWHM of two data points. Functional slices were co-registered to the anatomical data, and both data were normalized to Talairach space (Talairach and Tournoux, 1988).

Statistical analysis of the fMRI data was based on the general linear modeling (GLM) of the time series. For each subject, a design matrix was formed using a predictor for each experimental condition (“*Stereophonic-Voice*,” “*Stereophonic-Environment*,” “*Monophonic-Voice*,” “*Monophonic-Environment*”) and for the target blocks. The predicted time courses were adjusted for the hemodynamic response delay by convolution with a canonical hemodynamic response function (sum of two gamma functions).

Cortex-based realignment was performed for aligning the functional time series of individual subjects and to perform random effect group-based statistics (Goebel et al., 2006). Statistical maps were thresholded and corrected for multiple comparisons (alpha = 0.05) on the basis of cluster-level statistical threshold estimation performed on the cortical surface data (Forman et al., 1995; Goebel et al., 2006).

### MEG experiment and signal analysis

The sounds were presented with an interstimulus interval (from offset to onset) of 1500 ms in four separate ~6-min runs (“*Stereophonic-Voice*,” “*Stereophonic-Environment*,” “*Monophonic-Voice*,” “*Monophonic-Environment*”). Before each run, the subject was indicated verbally which stimulus attribute to attend to. The order of runs was counterbalanced across subjects.

Subjects were instructed to respond with a button press in case the attended sound attribute was the same in two consecutive sounds (10% of the cases). The target sounds were excluded from the analysis. The response hand was alternated across subjects.

The auditory evoked fields were recorded in a magnetically shielded room using a whole-head MEG systems with 275 axial gradiometers (VSM/CTF Systems Inc., Port Coquitlam, Canada; six subjects), and a 306-channel Vectorview device (Elekta Neuromag, Helsinki, Finland; four subjects). Head-position-indicator coils were attached to the scalp, and their positions were measured with a three-dimensional digitizer; the head coordinate frame was anchored to the two ear canals/periauricular points and the nasion. The head position was determined by feeding current to the marker coils and measuring their positions with respect to the sensory array.

The MEG signals were low-pass filtered at 300 Hz and digitized at 1200 Hz with the VSM/CTF Systems device, and band-pass filtered at 0.03–200 Hz and digitized at 600 Hz with the Vectorview system. The signals were averaged from 200 ms before the stimulus onset to 1000 ms after it, setting as baseline the 200-ms interval immediately preceding the stimulus onset. The averaged signals were digitally low-pass filtered at 40 Hz. The horizontal and vertical electro-oculograms were recorded to discard data contaminated by eye blinks and movements.

For source analysis, the head was modeled as a homogeneous spherical volume conductor. The model parameters were optimized for the intracranial space obtained from MR images that were available for all subjects. The neurophysiological responses were analyzed by first segregating the recorded sensor-level signals into spatiotemporal components, by means of manually-guided multi-dipole current modeling (equivalent current dipole, ECD; Hämäläinen et al., 1993). The analysis was conducted separately for each subject using Elekta Neuromag (Elekta Oy) software package, following standard procedures (Salmelin et al., 1994; Hansen et al., 2010). The parameters of an ECD represent the location, orientation, and strength of the current in the activated brain area. The ECDs were identified by searching for systematic local changes that persist for tens of milliseconds in the measured magnetic field pattern. ECD model parameters were then determined at those time points at which the magnetic field pattern was clearly dipolar. Only ECDs explaining more than 85% of the local field variance during each dipolar response peak were accepted in the multidipole model. Based on this criterion, 2–4 spatiotemporal components were selected into the individual subjects' models. The analysis was then extended to the entire time period, and all MEG channels were taken into account: The previously found ECDs were kept fixed in orientation and location while their strengths were allowed to change.

For optimizing the accuracy of the spatial fits, the orientation and location of the ECDs were estimated in each individual in the condition with the strongest signals in the time windows of the main experimental effects suggested by the sensor level data. To avoid spurious interactions between close-by sources of the 100-ms and sustained responses with similar current orientations, the dipoles modeled during the sustained responses were used to explain also the 100-ms responses. The variability in the signal-to-noise ratios between conditions was very small: Visual inspection and the calculated goodness-of-fit values obtained by comparing the original data and the data predicted by the fitted sources showed that the same sources explained well the responses in the other conditions as well.

The ECD source waveforms were analyzed for the late sustained (>300 ms) responses that were hypothesized to show the main experimental effects. Two measures on the strength of the response were obtained in each stimulus condition: (i) average over a 50-ms time window during the rising slope of the sustained response (360–410 ms; later in the text referred to as time window A), and (ii) average over a 100-ms time window centered at each individual's peak response, determined in the condition showing overall strongest signal (i.e., monophonic voice) and then applied in all experimental conditions (time window B). The response strengths were statistically tested using paired *t*-tests (two-sided, Bonferroni-corrected for multiple comparisons).

## Results

### Behavioral experiment

When presented separately, both environmental sounds and voices were recognized with high accuracy (99 ± 1%), whereas the reaction times were significantly longer for the environmental sounds than voices (931 ± 260 vs. 819 ± 250 ms, *P* < 0.01). When the sounds were superimposed, the sounds continued to be well recognizable, although subjects made more errors for the stereophonic than monophonic sounds (1.9 vs. 0.6%, *P* < 0.01) irrespective of whether attention was focused on the voice or environmental sound part. Reaction times did not significantly differ between the attended monophonic sounds (attention to environmental sounds 896 ± 260 ms vs. attention to voices 846 ± 290 ms, *P* = 0.06). In the stereophonic condition, reaction times were prolonged for environmental sounds but not for voices compared with the monophonic sounds (attention to environmental sounds 961 ± 270 ms, *P* < 0.01; attention to voices 840 ± 270 ms, *P* = 0.8; Attentional × Spatial condition interaction *P* = 0.05).

### FMRI results

**Figure 2:**
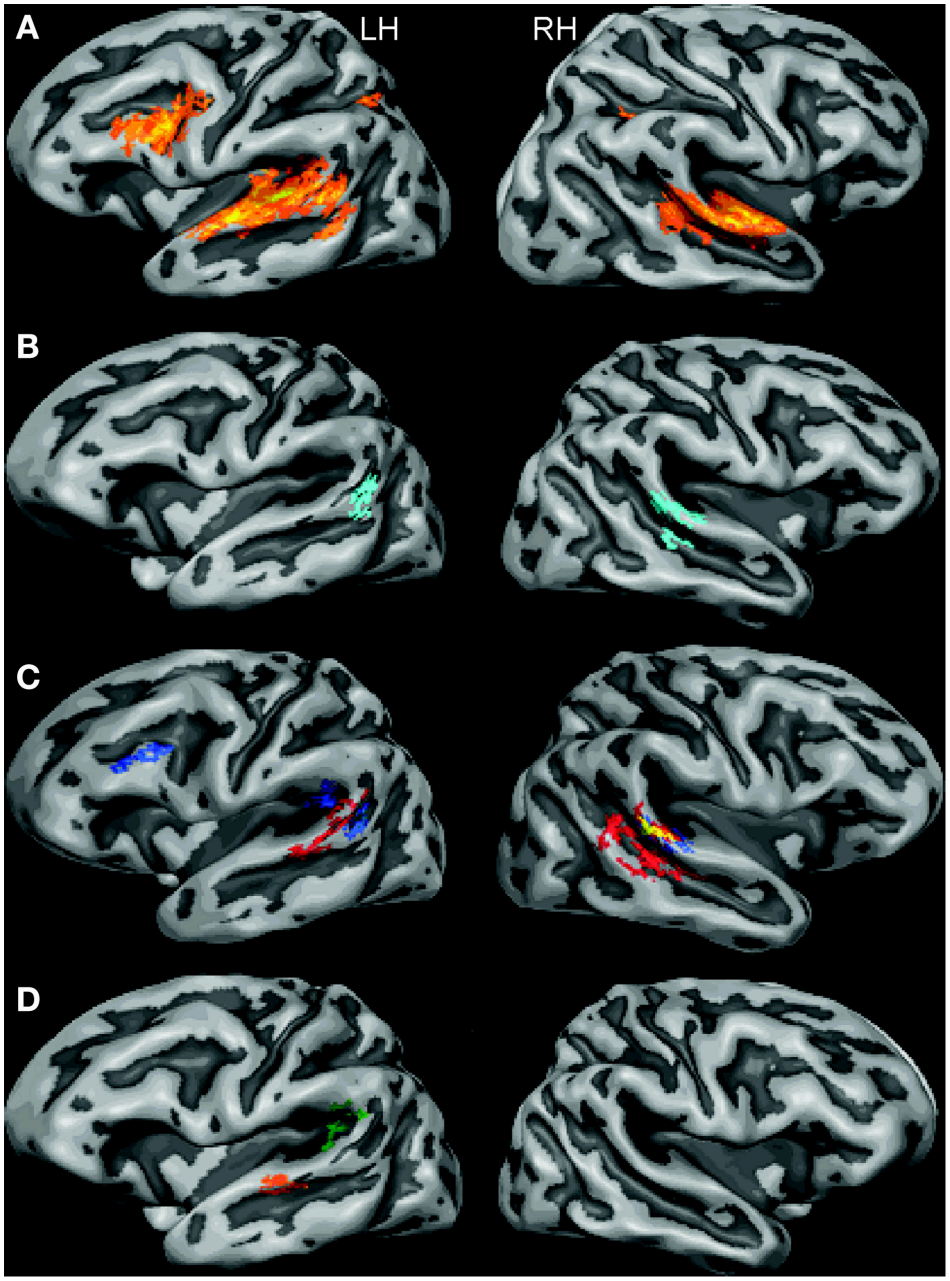
**Results from cortex-based aligned random effect analysis in stereophonic and monophonic experimental conditions (LH, left hemisphere; RH, right hemisphere). (A)** All conditions vs. baseline (F-map; the color ranges from yellow to reddish with increasing *f*-value). **(B)** “*Stereophonic*” vs. “*Monophonic”* stimuli (merged attention conditions; light blue: *Stereophonic* > *Monophonic*). **(C)** “*Stereophonic”* vs. “*Monophonic”* stimuli in the “*Voice”* condition (red: *Stereophonic-Voice* > *Monophonic-Voice*) and in the “*Environment”* condition (blue: *Stereophonic-Environment* > *Monophonic-Environment*); the overlapping area common for both these contrasts is highlighted in yellow. **(D)** The statistically significant interaction maps of the activations depicted in **(C)**. Green: “*Stereophonic-Environment*”—“*Monophonic-Environment*” > “*Stereophonic-Voice*”—“*Monophonic-Voice”*; orange: “*Stereophonic-Voice*”—*Monophonic- Voice”* > “*Stereophonic-Environment*”—“*Monophonic-Environment*.”

Listening to the auditory mini-scenes induced extensive activations at the superior temporal cortex bilaterally, including the Heschl's gyrus and surrounding regions at the superior temporal gyrus and sulcus (see Figure 2A). Additional activation was found in the left middle temporal gyrus (MTG), left inferior frontal gyrus (IFG), and bilateral inferior parietal lobule (IPL). The overall activation pattern was largely common to all experimental conditions.

#### “stereophonic” vs. “monophonic” scenes

To examine the brain regions especially involved in the processing of spatial cues, we first compared the activation to “*Stereophonic”* vs. “*Monophonic”* scenes grouped across the two attention conditions. We observed statistically significantly higher BOLD responses in the “*Stereophonic”* than “*Monophonic”* condition (see Figure 2B) bilaterally in the posterior-lateral STG regions. In the left hemisphere (LH), this region was located at the adjacency of the temporal-parietal border; in the right hemisphere (RH), an additional cluster was detected along the STS.

We further dissected the “*Stereophonic”* vs. “*Monophonic”* contrast by analyzing the two attention conditions separately, i.e., the two orthogonal contrasts' “*Stereophonic-Environment”* vs. “*Monophonic-Environment”* and “*Stereophonic-Voice”* vs. “*Monophonic-Voice*.” In the right posterior STG, these two contrasts were independently significant (marked with yellow in Figure 2C). Similarly, in the LH, clusters with significantly different responses in one of the two contrasts were interspersed, but in none of these locations both contrasts were independently significant. Besides these common clusters, activations specific to the different sound attributes the subjects were attending to were detected. When listeners attended to the environmental excerpts in the sounds, significant activation differences between “*Stereophonic”* and “*Monophonic”* conditions were found in the left PT and in the left IFG. Conversely, when listeners attended to the vocal excerpts in the sounds, significant activation differences between conditions were found in the left middle STG and in the right posterior and middle STS. These latter clusters resemble regions reported to be selectively activated for voices in previous studies (e.g., Belin et al., 2000; Bonte et al., 2014).

To test these observations statistically, interaction maps were calculated (Figure 2D). Of the regions for which any individual contrast was significant (blue or red regions in Figure 2C), only the regions in the left PT and in the left middle STG survived a rigorous statistical threshold (*p* < 0.05, corrected), whereas homologous activity in the right STS did not.

#### “voice” vs. “environment” scenes

To examine the brain regions specifically affected by the applied attentional manipulation, we compared the activations to the scenes grouped across the stereophonic and monophonic conditions. We observed significantly higher BOLD responses for the “*Environment*” condition in a largely left-lateralized network including posterior STG, posterior STS/MTG, and the dorsolateral prefrontal cortex (DLPFC). Bilateral activation of the posterior parietal cortex (PPC) and the left precentral gyrus (PrG) were also observed. No region showed increased activation for “*Voice*” condition compared with the “*Environment*” condition (See Supplementary Results). When analyzing the stereo- and monophonic conditions separately, i.e., “*Stereophonic-Environment”* vs. “*Stereophonic-Voice”* and “*Monophonic-Environment”* vs. “*Monophonic-Voice*,” a generally similar pattern of overall activation differences was observed.

### MEG results

**Figure 3:**
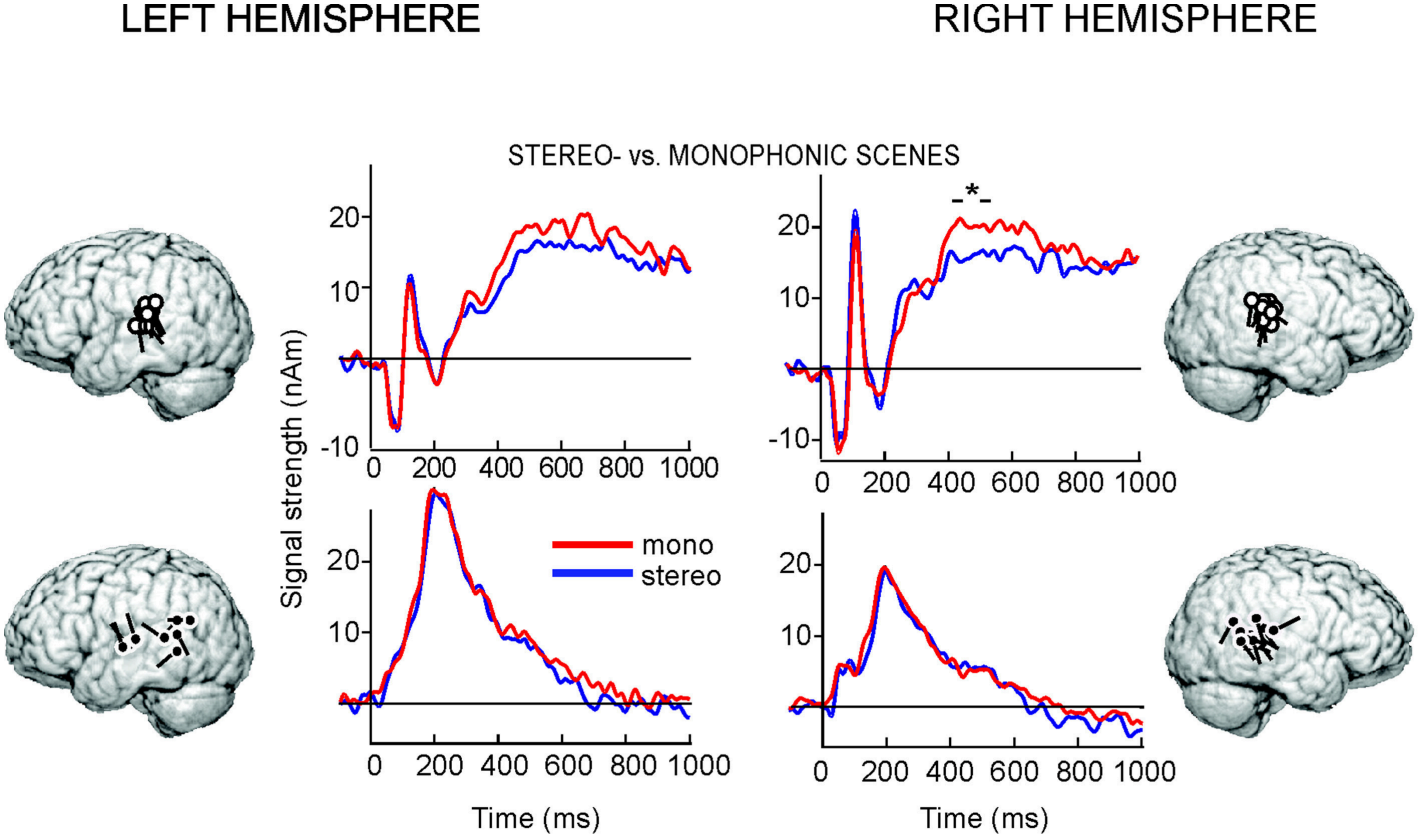
**MEG group-level data**. The locations and orientations of individual ECDs used to model the 100-ms, sustained (white dots), and 250-ms (black dots) responses superimposed on an average brain, and the corresponding averaged ECD time courses from −200 to 1000 ms with respect to the stimulus onset, grouped across the two attention conditions. The asterisk indicates the 100-ms time window with statistically significant difference (*P* < 0.04) between the conditions.

The initial sensor-level analysis revealed that all stimuli evoked strong responses bilaterally over the temporal areas, peaking at ~50, ~100, and at ~250–700 ms after the sound onset. In agreement with previous studies (for a review, see Hari, 1990), the prominent 100-ms responses were explained by two ECDs, one in the left (8 out of 10 subjects) and one in the right (10/10 subjects) supratemporal auditory cortex (individual source locations indicated by white dipoles in Figure 3). The same sources explained adequately also the 50-ms responses and the sustained activity peaking >300 ms.

In both hemispheres, another source with more variable location and direction of current flow over subjects was needed to explain the responses at ~250 ms (locations indicated by black dipoles in Figure 3; 8/10 and 10/10 subjects in the LH and RH, respectively), in line with earlier auditory MEG studies applying naturalistic sounds (Renvall et al., 2012a,b).

The activation strengths and latencies were fairly similar in all stimulus conditions (see Figure 3). We first compared the activation to “*Stereophonic*” vs. “*Monophonic*” scenes grouped across the two attention conditions. In the RH, sustained responses peaking at ~465 ± 20 ms (time window B, mean ± SEM over subjects) were stronger in the “*Monophonic*” than “*Stereophonic*” condition (*P* < 0.04). The responses did not statistically significantly differ between conditions during the response rise time 360–410 ms (time window A) in the RH (*P* = 0.09), nor in either of the tested analysis time windows in the LH (time window A, *P* = 0.07; time window B, LH peak ~535 ± 30 ms, *P* = 0.18).

**Figure 4:**
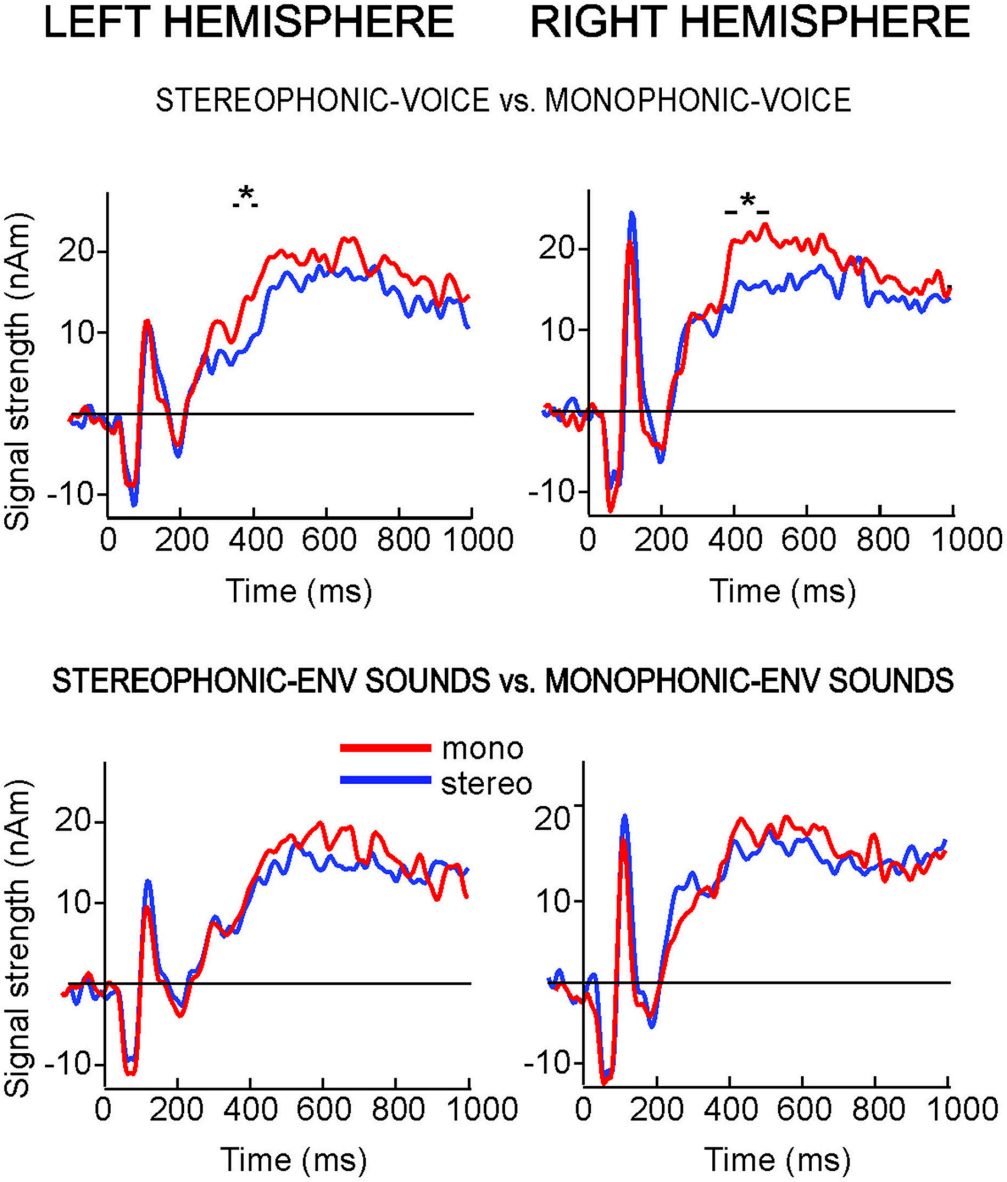
**Time courses of the ECDs used to model the 100-ms and sustained responses to stereo- vs. monophonic sounds in both attentional conditions**. (**Top**: attention to voice; **bottom**: attention to environmental sounds). The asterisks indicate the 50-ms (LH) and 100-ms time windows (RH) with statistically significant differences between the conditions.

When the two attention conditions were analyzed separately (Figure 4), the “*Monophonic*” scenes produced stronger activation than “*Stereophonic”* scenes only when the subjects were attending to voices (“*Stereophonic-Voice”* vs. “*Monophonic-Voice”*: RH time window B, *P* < 0.03; LH time window A, *P* < 0.02). The 250-ms responses did not differ between the stimulus conditions.

Finally, we examined the effects of the applied attentional manipulation by comparing the activations to scenes grouped across the stereophonic and monophonic conditions (“*Voice*” vs. “*Environment*” scenes). For this comparison, no significant effect of condition was detected in either hemisphere.

## Discussion

The present study investigated cortical processing of complex, naturalistic auditory mini-scenes with (stereophonic), or without (monophonic) spatial acoustic information. In particular, modulating the focus of subjects' attention between superimposed human voices and other natural sounds enabled us to study auditory cortical mechanisms related to selecting sound objects from real-life-like auditory scenes.

Listening to stereophonic scenes resulted in a robust increase of BOLD response in the right and the left posterior auditory cortex. This increased activation was present irrespective of whether the subjects were attending to voices or environmental sounds. The location of task-independent activation corresponded to the posterior portion of planum temporale, a site compatible with cytoarchitectonic area Tpt (Galaburda and Sanides, 1980; Sweet et al., 2005), area STA (Rivier and Clarke, 1997; Wallace et al., 2002), or area Te3 (Morosan et al., 2005). These locations are also in agreement with previous functional neuroimaging studies that have investigated sound localization and motion using sounds presented in isolation (Hart et al., 2004; Krumbholz et al., 2005a; Callan et al., 2013; Derey et al., 2016). Our results with real-life-like auditory stimuli support the role of these areas in processing auditory spatial information, and, in line with a “where” auditory processing stream, point to their role in the analysis of spatial cues also within complex auditory scenes. Processing of spatial acoustic cues in these areas seems to be rather automatic and only marginally influenced by subjects' focus of attention, which may be particularly relevant for efficient localization of relevant sounds. It is worth noting that our experimental task did not explicitly require listeners to localize the sounds, which further highlights the obligatory nature of the observed effects.

Besides sound localization, spatial acoustic cues within complex auditory scenes contribute to sound stream segregation and formation (Bregman, 1990). Thus, any effects related to the focus of auditory attention during the listening task may reflect cortical processing mechanisms devoted to integration of spatial cues with other spectral and temporal cues, with the ultimate goal of segregating and grouping relevant sound objects in a scene. The present results are consistent with this view. The fMRI BOLD responses in the left STG and the right STS, as well as the late sustained MEG responses from ~360 ms onwards bilaterally, were affected by the spatial sound manipulation when voice attributes within the stimuli were attended to. The observed effect is unlikely to be related to greater demands in attending to stereophonic vs. monophonic voice sounds since the reaction times to stereophonic and monophonic sounds were similar for voices but differed for environmental sounds. Moreover, the anatomical locations of the BOLD responses resemble the so-called “voice-sensitive” regions reported in previous studies (Belin et al., 2000).

Interestingly, the BOLD responses at these areas were larger for the stereophonic sounds, whereas the MEG responses showed the opposite effect with stronger responses to monophonic sounds. The MEG results are in concordance with an earlier observation of decreased auditory steady-state responses for pseudo-stereophonic vs. monophonic sounds (Ross et al., 2004). MEG evoked responses are highly sensitive to the synchronicity of neuronal activation, and the current results are likely to reflect a reduced convergence of temporally coincident neural firing during the stereophonic stimulation with different acoustic inputs to the two ears compared with the monophonic stimulus presentation. In addition, active neuronal inhibition in the auditory cortex (Fitzpatrick et al., 1997) could result in a simultaneous increment of BOLD responses and decrement of MEG responses.

Furthermore, we observed an effect of spatial acoustic cues on the BOLD response at the left IFG and PT when attention was directed to environmental sounds. PT has previously been associated with processing of tool sounds (Lewis et al., 2005), which in fact constitute a large subset of our experimental sounds. In contrast, our MEG experiment did not reveal a time window nor source areas with similar effect. This discrepancy between fMRI and MEG responses can be at least partly related to MEG's suboptimal sensitivity to spatially extended frontal activations (Tarkiainen et al., 2003).

Although consistent with previous studies, our interpretations above are not univocal. In our scenes, voices were located somewhat more centrally for mimicking typical communicational situations, while the environmental sounds were more variable in their original left-right channel difference. However, as the MEG responses differed between mono- and stereophonic conditions only when attending to voices, the effect is unlikely to be related to purely acoustical differences between the attended sounds, as such differences between the conditions were actually slightly larger for the environmental than voice sounds. Further studies could verify whether the experimental effects that were dependent on the focus of attention, indeed, reflect mainly the different sensitivity of the outlined regions to the distinct sound categories, or relate to the variation of different acoustic cues within the auditory scene as well.

When the subjects focused their attention on the “Environment” excerpts of the sounds irrespective of the spatial aspects of the sound, a robust increase of fMRI BOLD in the left temporal, left frontal, and bilateral parietal areas was detected compared with attending to “Voices,” whereas neither fMRI nor MEG responses highlighted areas or time windows with greater activity for “Voices.” This result can be interpreted in the light of ecological validity and/or acoustic properties of the corresponding stimuli. Attending to the environmental sounds within our scenes may have required additional top-down signaling from frontal and parietal areas for overriding or counteracting the automatic allocation of attention to vocalized sounds. This interpretation is supported by the longer reaction times to environmental sounds in the behavioral experiment.

Here we used measures of neural activation that are most frequently used in neurophysiological and hemodynamic non-invasive brain mapping, namely, MEG evoked responses and fMRI BOLD signals. Other measures could be more sensitive to such fine-grained changes in brain activations as examined in the present study. Indeed oscillatory activity, especially in the high-gamma range (>75 Hz) measured intracortically from the auditory cortices (Mesgarani and Chang, 2012; Zion Golumbic et al., 2013) has been demonstrated to track the envelope of attended speech within multi-talker settings.

Several modeling studies have recently successfully mapped different tempo-spectral characteristics of acoustic stimuli to their corresponding neural representations (Pasley et al., 2012; Mesgarani et al., 2014; Santoro et al., 2014), suggesting strong correspondences between the auditory input and cortical reactivity. However, in real life the input to the ears is often a complicated mixture from several sound sources, thus requiring auditory cortical areas to use more sophisticated computational scene-analysis strategies for sound localization than the pure physical cue extraction (Młynarski and Jost, 2014). Accordingly, the current results show that spatial auditory cues—already within rather simple, but naturalistic auditory stimuli—are processed together with other relevant temporal and spectral cues, and that the related cortical processing is attention- and stimulus-dependent.

## Author contributions

HR and NS contributed equally to this work. HR, NS, and EF designed the experiment. HR and NS recorded the stimuli and acquired the fMRI and MEG data. CB and AL ran and analyzed the behavioral experiment. HR, NS, and EF analyzed the imaging data. HR, EF, and NS wrote the paper.

## Funding

The authors declare no conflicting interests. Funding for the present research was contributed to EF from the Netherlands Organization for Scientific Research (NWO, Vernieuwingsimpuls VIDI 452-04-330, VICI 453-12-002) and from the Province of Limburg and to HR from the Academy of Finland (grants #127401 and #277655).

### Conflict of interest statement

The authors declare that the research was conducted in the absence of any commercial or financial relationships that could be construed as a potential conflict of interest.
