# Supplementary material for: Attention Modulates the Auditory Cortical Processing of Spatial and Category Cues in Naturalistic Auditory Scenes
Source: Front Neurosci. 2016 Jun 7;10:254. doi: 10.3389/fnins.2016.00254 (PMC4894904; doi:10.3389/fnins.2016.00254)
Supplement: Supplementary file 1 [file Image1.PDF]

Supplementary Results

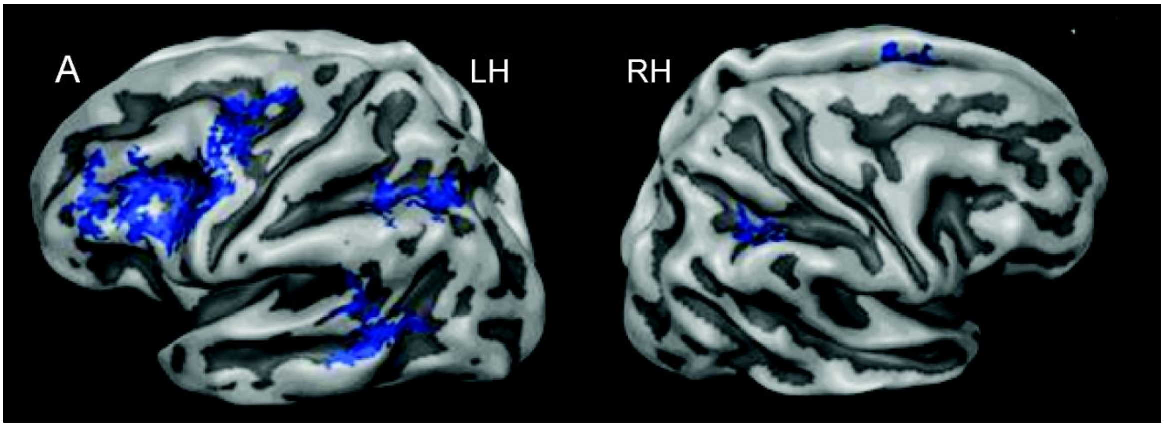

Results from cortex-based aligned random effect analysis for environmental sound versus voice conditions (LH = left hemisphere, RH = right hemisphere). a) '*Environment*' versus '*Voice'* contrast (merged spatial conditions); blue: *Environment* > *Voice*
